# Long-term tolerability and maintenance of therapeutic response to sodium oxybate in an open-label extension study in patients with fibromyalgia

**DOI:** 10.1186/ar4375

**Published:** 2013-11-11

**Authors:** Michael Spaeth, Cayetano Alegre, Serge Perrot, Youyu Grace Wang, Diane R Guinta, Sarah Alvarez-Horine, Irwin Jon Russell

**Affiliations:** 1 Rheumatologische Schwerpunktpraxis, Bahnhofstrasse 95, 82166 Graefelfing, Munich, Germany; 2 Institut Universitari Dexeus, Carrer Sabino de Arana 5, 08028 Barcelona, Spain; 3 Service de Médecine Interne et Consultation de la Douleur, Hôpital Dieu, Université Paris, Descartes, INSERM U 987, 1 Place du Parvis Notre Dame, 75004 Paris, France; 4 Jazz Pharmaceuticals, Inc, 3180 Porter Drive, Palo Alto, CA 94304, USA; 5 Department of Medicine, University of Texas Health Science Center at San Antonio, 7434 Floyd Curl Drive, San Antonio, TX 78229, USA; 6 Leitender Arzt Rheumatologie, Spital Linth, 8730 Uznach, Switzerland

## Abstract

**Introduction:**

The long-term safety and therapeutic response of sodium oxybate (SXB) in fibromyalgia syndrome (FM) patients were assessed for a combined period of up to 1 year in a prospective, multicenter, open-label, extension study in patients completing 1 of 2 phase 3 randomized, double-blind, controlled, 14-week trials that examined the efficacy and safety of SXB 4.5 g, SXB 6 g, and placebo for treatment of FM.

**Methods:**

This extension study comprised an additional 38 weeks of treatment and was carried out at 130 clinical sites in 7 countries. Initial entry criteria for the previous 2 double-blind clinical trials required that patients aged ≥ 18 years met the American College of Rheumatology 1990 criteria for FM, had a body mass index (BMI) < 40 kg/m^2^, and had a score ≥ 50 on a 100-mm pain visual analog scale (VAS) at baseline. All patients began treatment in the extension study with SXB 4.5 g/night (administered in 2 equally divided doses) for at least 1 week, followed by possible serial 1.5 g/night dose increases to 9 g/night (maximum) or reductions to 4.5 g/night (minimum).

**Results:**

Of the 560 FM patients enrolled in this extension study, 319 (57.0%) completed the study. The main reason for early discontinuation was adverse events (AEs; 23.0% of patients). Patients were primarily middle-aged (mean 46.9 ± 10.8 years), female (91.1%), white (91.4%), with a mean duration of FM symptoms of 9.9 ± 8.7 years. Serious AEs were experienced by 3.6% of patients. The most frequently reported AEs (incidence ≥ 5% at any dose or overall) were nausea, headache, dizziness, nasopharyngitis, vomiting, sinusitis, diarrhea, anxiety, insomnia, influenza, somnolence, upper respiratory tract infection, muscle spasms, urinary tract infection, and gastroenteritis viral. Maintenance of SXB therapeutic response was demonstrated with continued improvement from controlled-study baseline in pain VAS, Fibromyalgia Impact Questionnaire (FIQ) total scores, and other measures. Responder analyses showed that 68.8% of patients achieved ≥ 30% reduction in pain VAS and 69.7% achieved ≥ 30% reduction in FIQ total score at study endpoint.

**Conclusions:**

The long-term safety profile of SXB in FM patients was similar to that in the previously reported controlled clinical trials. Improvement in pain and other FM clinical domains was maintained during long-term use.

**Trial registration:**

ClinicalTrials.gov NCT00423605.

## Introduction

Fibromyalgia syndrome (FM) is a multidimensional disorder with many clinical manifestations. Consequently, it has been challenging to characterize its etiology and to identify a single treatment that addresses all of its manifestations. The 1990 American College of Rheumatology (ACR) criteria for FM focused on chronic widespread pain and tenderness at 11 or more of 18 tender points [1]. By design, the recent 2010 ACR diagnostic criteria integrated widespread pain with other important clinical domains [2] and abandoned tender-point examination. The 2010 ACR criteria identify combinations of clinical features, including chronic widespread pain, sleep disturbance, fatigue, and dyscognition [2-6]. The move to involve domains other than pain in these criteria is in line with the 2009 OMERACT (Outcome Measures in Rheumatoid Arthritis Clinical Trials) international guidelines for important research domains in fibromyalgia studies [7,8], which also included low pain threshold (allodynia), psychological factors (anxiety and depression) and physical function. Additionally, OMERACT worked with physicians and FM patients to develop consensus regarding core symptom domains that should be assessed in FM clinical trials, and screening tools have also been developed with the same methodology [9]. These core domains include pain, tenderness, sleep disturbance, fatigue, patient global assessment, and multidimensional function [7,8].

Only a few medications have demonstrated efficacy relative to placebo in reducing pain, but none have shown efficacy across all outcomes including functional impairment, fatigue, sleep disturbance and quality of life (QoL). The United States (US) Food and Drug Administration approved pregabalin, duloxetine, and milnacipran for the treatment of FM [10-12] based on randomized clinical trials lasting up to six months. Extension studies of all three drugs have further suggested that long-term tolerability and efficacy are consistent with that observed in the clinical trials [13-17]. Amitriptyline has been widely prescribed for FM and is recommended across existing FM treatment guidelines [18] but it was never formally evaluated by US or European Union (EU) regulators for FM. While a recent meta-analysis suggested that amitriptyline was superior to duloxetine and milnacipran in improving pain, sleep disturbances, fatigue and QoL in FM at minimum dosages of 10 and 50 mg/day, the methodological quality of the amitriptyline studies was considered poor [19], and tachyphylaxis has been reported to arise in less than three months [19,20]. Furthermore, meta-analyses of currently approved medications have shown only modest efficacy for pain and have not shown efficacy on other domains [19,21,22], and population studies have not demonstrated that FM medications have had any meaningful effect on outcomes over time [23].

Sodium oxybate (SXB) is the sodium salt of γ-hydroxybutyrate (GHB), an endogenous metabolite of γ-aminobutyric acid (GABA) with central nervous system (CNS) depressant properties. As an oral solution, SXB (Xyrem®) is approved in the US, the EU and Canada for treating cataplexy and various symptoms in patients with narcolepsy [24-26]. SXB taken orally (Alcover®) is approved in the EU for treatment of alcohol withdrawal and, as an intravenous adjuvant (Gamma-OH™, Somsanit®), is also approved as a sedating anesthetic. Because of its ability to modify sleep in narcolepsy, the effects of SXB on sleep physiology have been studied. SXB increased slow-wave sleep (SWS) and reduced sleep fragmentation in patients with narcolepsy [27,28]. A phase 2 study in FM patients reported that treatment for eight weeks with SXB 4.5 and 6 g/night decreased rapid eye movement (REM) sleep and improved morning fatigue, and that the 6-g dose also improved afternoon, evening, and overall fatigue; reduced wakefulness after sleep onset; and increased SWS and total non-REM sleep compared with placebo [29]. In the past few years, SXB has been evaluated in several placebo-controlled clinical trials for the treatment of FM [30-33]; however, it is not approved for this indication in the US, the EU, or in Canada.

Beneficial effects of SXB in the treatment of FM were demonstrated in two 14-week, phase 3, multicenter, placebo-controlled studies with 548 and 573 patients, respectively [32,33]. In these trials, patients were given SXB 4.5 or 6 g/night or placebo in two doses (at bedtime and 2.5 to 4 hours later). SXB was shown to be effective for two primary clinically relevant efficacy endpoints: pain severity, defined as the proportion of patients achieving ≥30% reduction on the pain visual analog scale (VAS) from baseline to week 14; and functionality, defined as the proportion of patients achieving ≥30% reduction in the Fibromyalgia Impact Questionnaire (FIQ) total score from baseline to week 14. A 30% reduction in pain is recommended as a clinically relevant outcome in chronic pain trials [34], and a 14% change on the FIQ is considered clinically relevant [35]. Improvements in patient-reported outcomes of fatigue, patient global impression of change (PGI-c), 36-item Short Form Health Survey (SF-36) physical component summary (PCS), and Jenkins Sleep Scale (for sleep quality) were also demonstrated.

The adverse-event (AE) profiles observed with SXB in these studies were similar to those reported with SXB in patients with narcolepsy [24]. There were no deaths, and the most common AEs in SXB-treated patients, with an incidence ≥5% and twice that of placebo in both studies, were nausea, dizziness, vomiting, and anxiety [32,33].

Given the chronic nature of FM, it is important to evaluate the long-term safety of SXB in patients with FM and to address questions about the duration of therapeutic response. The current open-label extension study was designed to assess the long-term safety and therapeutic response of SXB for a combined period of up to one year in patients completing the phase 3 placebo-controlled trials of SXB (14 weeks double-blind plus 38 weeks open-label extension study treatment) [32,33]. The long-term effects of SXB on QoL and daytime fatigue in patients with FM were also assessed.

## Methods

### Study design and patient selection

This prospective, multicenter, open-label extension study of SXB for the treatment of FM was carried out at 130 sites (106 sites in the US, 7 in Germany, 6 in France, 6 in Spain, 3 in the United Kingdom, and 1 each in The Netherlands and Italy) between January 2007 and January 2010. The study was approved by the following institutional review boards (IRBs) or ethics committees: Quorum Review IRB (Seattle, WA, USA); The University of Texas Health Science Center IRB (San Antonio, TX, USA), Western IRB (Olympia, WA, USA), Research Development and Administration IRB (Portland, OR, USA), Riverside Research Ethics Committee (London, UK), Hospital General Gregorio Marañón Ethics Committee (Madrid, Spain), Bayerische Landesärztekammer Ethics Committee (Munich, Germany), Azienda Ospedaliero-Universitaria Pisana Ethics Committee (Rome, Italy), Medisch Spectrum Twente Ethics Committee (Enschede, The Netherlands), and Hôpital Hôtel-Dieu Ethics Committee (Paris, France). Each patient provided informed consent prior to study initiation. The study was conducted in accordance with the Declaration of Helsinki and Good Clinical Practice Guidelines.

Patients with FM who completed either of the two phase 3, placebo-controlled, double-blind, 14-week trials (studies 06–008 [32] and 06–009 [33]) evaluating the efficacy and safety of SXB 4.5 g/night and SXB 6 g/night were eligible to enroll in this open-label extension study designed to assess the long-term safety and maintenance of efficacy of SXB. Within seven days of completing the phase 3 double-blind clinical trial, patients were enrolled in the extension study to receive up to 38 additional weeks of treatment, followed by two weeks of post-treatment follow-up. The total combined duration of treatment (double-blind phase and open-label extension) was up to 52 weeks. For consistency in this analysis, weeks of therapy are numbered to reflect the total combined exposure: weeks 1 to 14 were the phase 3 double-blind period, and ‘baseline’ refers to the baseline at the start of the double-blind period.

### Treatments

Regardless of their prior allocation and SXB dose during phase 3 double-blind treatment, all patients entering the long-term extension were initiated on SXB 4.5 g/night, administered in two equally divided doses and remained at that dose level for at least one week. Subsequent dosage increases to achieve therapeutic response were permitted in increments of 1.5 g/night at intervals of at least one week, to a maximum dose of SXB 9 g/night. Dosage reductions to address safety and tolerability were permitted in multiples of 1.5 g/night at intervals of any duration, to a minimum dose of SXB 4.5 g/night. Patients unable to tolerate SXB 4.5 g/night were discontinued from the study. Individual dose titration was based on the investigator’s impression of clinical effect and tolerability.

### Safety evaluations

The safety population consisted of all patients who received at least one dose of SXB. Safety was assessed by the incidence of AEs including serious AEs (SAEs), clinical laboratory values, suicidality and depression assessments, electrocardiograms, vital signs and physical examination findings, including body weight. All observed or spontaneously reported treatment-emergent AEs were assessed by prespecified criteria for severity and seriousness, and recorded, along with findings from physical examinations, including vital signs and a 12-lead electrocardiogram (obtained at week 52 of the combined double-blind and extension periods). AEs were coded by the Medical Dictionary for Regulatory Activities (MedDRA, Version 9.1). To assess risk of suicidality and occurrence of major depression, portions of the Mini-International Neuropsychiatric Interview (MINI) [36] were administered at prespecified time points, as was the Beck Depression Inventory version II (BDI-II) [37], in accordance with recommendations from IMMPACT (Initiatives on Methods, Measurement, and Pain Assessment in Clinical Trials) [34].

### Clinical evaluations

Clinical evaluations included overall pain severity, assessed by a pain VAS, and fatigue, also assessed by a VAS; both were administered every evening in an electronic diary. The VAS was a 5-cm line, with measurements converted to the equivalent of a 100-mm VAS for analysis (0 = no pain/fatigue to 100 = worst imaginable pain/fatigue). The mean changes in VAS scores from double-blind baseline values for pain and fatigue were determined weekly in the extension study from weeks 14 to 26, and monthly from weeks 30 to 50; week 52 was also assessed. The proportions of patients who achieved ≥30% and ≥50% reductions in overall pain VAS from baseline to extension-study endpoint were also determined in accordance with recommendations for chronic-pain trials [34]. The FIQ [38], PGI-c, and clinical global impression of change (CGI-c) [39] were performed at weeks 16, 18 and 22, and at the monthly visits during weeks 26 to 52. The FIQ, which is a patient self-report questionnaire that evaluates the impact of FM on daily life, has 10 subscales (physical impairment, did not feel good, work missed, difficulty with work, pain, fatigue, tired upon awakening, stiffness, anxiety and depression). The score for each subscale ranges from 0 to 10. An FIQ total score is the sum of the scores from the 10 subscales and it ranges from 0 to 100, with higher scores indicating greater impact of FM on functioning; a decrease in score demonstrates improvement. The proportion of patients who achieved ≥30% reduction in FIQ total score from double-blind baseline to extension-study endpoint was also determined. The PGI-c asked patients to rate their FM since they started blinded SXB treatment on a 7-point scale (very much better, much better, a little better, no change, a little worse, much worse, and very much worse); thus, there was no baseline assessment for this endpoint. Responders for PGI-c were characterized by the proportion of patients who reported their FM as ‘very much better’ or ‘much better.’ The CGI-c was the investigator’s assessment of change from baseline in the patient’s overall FM condition; the CGI-c was analyzed as the proportion of patients rated by the investigator as ‘very much improved’ or ‘much improved.’ The SF-36 [40] assessed the patient’s QoL at weeks 18, 38, and 52; an increase in score indicates improvement. Specifically, physical functioning was assessed using the SF-36 PCS score, a prespecified secondary endpoint in the double-blind studies. The mental component summary (MCS) of the SF-36 was also evaluated.

The proportion of patients meeting composite response measures was also determined. The FM Syndrome Composite Response was predefined as the proportion of patients who achieved ≥30% reduction in pain VAS, a PGI-c response of ‘much better’ or ‘very much better,’ and ≥30% reduction in FIQ total score. The FM Pain Composite Response was the proportion of patients who achieved ≥30% reduction in pain VAS and a PGI-c response of ‘much better’ or ‘very much better.’

Changes from double-blind baseline in the FIQ ‘tired upon awakening’ subscale and in the Functional Outcomes of Sleep Questionnaire (FOSQ) [41] were determined at extension-study endpoint. The FOSQ is a patient-reported questionnaire that evaluates the effects of excessive sleepiness on daytime functioning; lower scores indicate greater difficulty in daily functioning.

### Statistical analysis

All data were summarized using descriptive statistics. Analyses of changes in safety and efficacy parameters were relative to the double-blind baseline, with study endpoint defined as the last available data at extension-study completion (week 52) or early discontinuation.

Data in summary tables (except AEs) are presented by final dose in the extension study; the final dose was defined as the last dose prescribed for the patient by the investigator. Data in AE summary tables are presented by the last dose taken at the time of event onset.

## Results

### Patient disposition and baseline demographics

**Figure 1:**
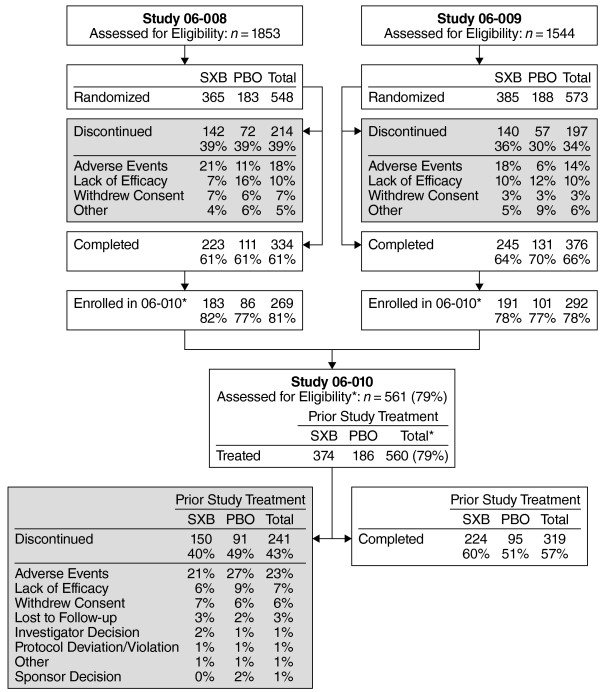
**Disposition of patients.** Studies 06–008 and 06–009 were concurrent phase 3, double-blind, placebo-controlled, clinical trials; 06–010 is the open-label extension of the phase 3 trials. The denominator for studies 06–008 and 06–009 is the number of randomized patients, and the denominator for 06–010 is the number of treated patients. *Denominator is the number of completed patients from 06–008/06–009. PBO, placebo; SXB, sodium oxybate.

Of the 710 patients who completed double-blind treatment, 561 patients (79%) were assessed for eligibility in the long-term open-label extension study, and 560 patients were treated (Figure 1); one patient was not entered into the extension trial because of a positive test for cannabinoids on the drugs of abuse screen. The percentages of patients who entered the long-term extension from each of the two controlled studies were similar (81% for Study 06–008 and 78% for Study 06–009).

Overall, 319 (57%) patients completed the study, including 51% of those who had taken placebo and 60% of those who had taken SXB during the double-blind period. The most common reasons for discontinuation were AEs (23%, including non–treatment-emergent and treatment-emergent AEs), lack of efficacy (7%), withdrawal of consent (6%), and lost to follow-up (3%; Figure 1). The mean length of exposure was 198.9 nights.

**Figure 2:**
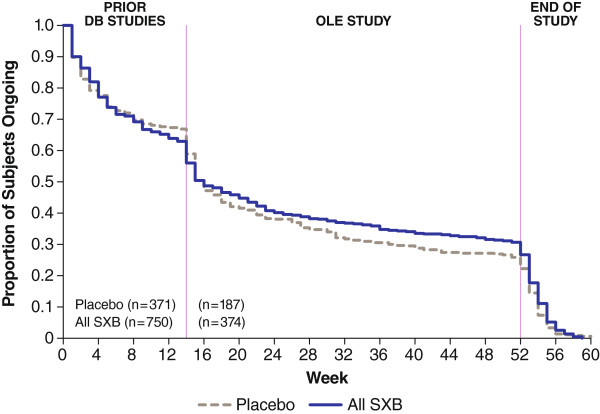
Kaplan-Meier survival curve of subjects in studies 06–008 and 06–009 pooled, stratified, and summarized by double-blind treatment group and sequentially linked with data from subjects continuing into the open-label extension.

Figure 2 is a Kaplan-Meier survival curve of patient retention throughout all phases of the study and presents final retention rates with respect to participants who were randomized to each of the treatment groups during the double-blind phase as well as for subjects who subsequently enrolled in the open-label extension. As shown, extension study discontinuations were greater among patients who had been randomized to placebo in the parent study and were SXB-naïve prior to the extension, with these discontinuations mainly due to AEs and lack of efficacy.

Patients were predominantly female (91.1%) and white (91.4%), with a mean ± standard deviation (SD) age of 46.9 ± 10.8 years and mean ± SD body mass index (BMI) of 28.4 ± 4.6 kg/m^2^. The mean ± SD duration of FM symptoms was 9.9 ± 8.7 years (Table 1), and nearly all patients (97.5%) had received treatment for FM prior to their participation in the double-blind studies.

**Table 1:** Demographic and clinical characteristics of patients enrolled in the open-label extension

**Parameter**	**Value (Number = 560)**
Age, years	
Mean (SD)	46.9 (10.8)
≥ 65 years, number (%)	23 (4.1)
Sex, number (%)	
Male	50 (8.9)
Female	510 (91.1)
Race, number (%)	
White	512 (91.4)
Black	34 (6.1)
Asian	6 (1.1)
Other	8 (1.4)
BMI, kg/m^2^	
Mean (SD)	28.4 (4.6)
Range	16.4–41.5
Years since first FM symptoms	
Mean (SD)	9.9 (8.7)
Double-blind baseline values, mean (SD)	
Pain VAS	72.0 (13.3)
Fatigue VAS	73.1 (14.8)
FIQ total score	62.4 (14.1)

BMI, body mass index; FIQ, Fibromyalgia Impact Questionnaire; FM, fibromyalgia syndrome; SD, standard deviation; VAS, visual analog scale.

Double-blind baseline values for patients who entered the extension study indicated the presence of moderate to severe pain (mean ± SD pain VAS of 72.0 ± 13.3) and fatigue (mean ± SD fatigue VAS of 73.1 ± 14.8), and impaired functionality in daily living (mean ± SD FIQ total score of 62.4 ± 14.1; Table 1).

### Long-term safety

No deaths occurred during the study. Overall, 498 of 560 (88.9%) treated patients reported at least one treatment-emergent AE. The overall incidences of AEs for the SXB 4.5-g, 6-g, 7.5-g and 9-g dose groups were 54.1%, 61.3%, 68.2% and 78.6%, respectively (Table 2). The most frequently reported AEs (incidence ≥5% at any dose or overall) were nausea, headache, dizziness, nasopharyngitis, vomiting, sinusitis, diarrhea, anxiety, insomnia, influenza, somnolence, upper respiratory tract infection, muscle spasms, urinary tract infection and gastroenteritis viral (Table 2). The maximum severity of most AEs was assessed as mild or moderate in all dose groups. The proportion of patients who discontinued due to a treatment-emergent AE was 21.8% (122/560 patients), and the proportions were similar among the dose groups: 4.5 g, 10.5%; 6 g, 8.3%; 7.5 g, 10.0%; and 9 g, 8.9%. The most frequent treatment-emergent AEs leading to study discontinuation were nausea (3.2%), anxiety (2.3%) and dizziness (2.0%). Of patients who discontinued due to AEs, 55.8% had done so by the end of month 2 in the extension study.

**Table 2:** Most frequent treatment-emergent AEs by dose at time of AE onset in safety population

**AE**	**Sodium oxybate dose at event onset**^**a**^				
	**4.5 g (n = 545)**	**6 g (n = 432)**	**7.5 g (n = 239)**	**9 g (n = 112)**	**Total (N = 560)**^**b**^
Any AE, number (%)^c^	295 (54.1)	265 (61.3)	163 (68.2)	88 (78.6)	498 (88.9)
Nausea	44 (8.1)	44 (10.2)	23 (9.6)	10 (8.9)	112 (20.0)
Headache	42 (7.7)	43 (10.0)	21 (8.8)	7 (6.3)	106 (18.9)
Dizziness	29 (5.3)	28 (6.5)	13 (5.4)	6 (5.4)	72 (12.9)
Nasopharyngitis	25 (4.6)	21 (4.9)	7 (2.9)	6 (5.4)	57 (10.2)
Vomiting	21 (3.9)	16 (3.7)	9 (3.8)	7 (6.3)	52 (9.3)
Sinusitis	14 (2.6)	21 (4.9)	13 (5.4)	4 (3.6)	50 (8.9)
Diarrhea	16 (2.9)	24 (5.6)	7 (2.9)	4 (3.6)	49 (8.8)
Anxiety	18 (3.3)	18 (4.2)	11 (4.6)	3 (2.7)	44 (7.9)
Insomnia	12 (2.2)	14 (3.2)	10 (4.2)	3 (2.7)	38 (6.8)
Influenza	12 (2.2)	14 (3.2)	9 (3.8)	3 (2.7)	37 (6.6)
Somnolence	10 (1.8)	15 (3.5)	4 (1.7)	7 (6.3)	34 (6.1)
Upper respiratory tract infection	2 (0.4)	18 (4.2)	11 (4.6)	4 (3.6)	34 (6.1)
Muscle spasms	7 (1.3)	8 (1.9)	10 (4.2)	4 (3.6)	29 (5.2)
Urinary tract infection	5 (0.9)	8 (1.9)	5 (2.1)	6 (5.4)	22 (3.9)
Gastroenteritis viral	1 (0.2)	4 (0.9)	5 (2.1)	6 (5.4)	16 (2.9)

‘Most frequent’ is defined as ≥5% in any dose group or overall. ^a^All patients began treatment in the long-term extension study with SXB 4.5 g/night and remained at that dose level for at least one week, with subsequent dose adjustments to address the level of response as well as safety and tolerability as stated in ‘Methods;’ ^b^if the dose at AE onset was not among the indicated doses, the patient was summarized in the ‘Total’ group only; ^c^values of Any AE, (number and percent) may not match the sum of the listed individual events since uncommon events are not included. AE, adverse event.

Twenty (3.6%) patients experienced at least one treatment-emergent SAE: seven (1.3%), five (1.2%), two (0.8%) and seven (6.3%) patients in the SXB 4.5-g, 6-g, 7.5-g and 9-g groups, respectively (one patient experienced SAEs at two different doses). These SAEs were single events unless otherwise noted and included atrial fibrillation; abdominal pain; upper abdominal pain; gastrointestinal hypomotility; chest pain in two patients; cholelithiasis in two patients with chronic cholecystitis in one of those patients and an additional case of acute cholecystitis; *Clostridium difficile* colitis and endometriosis; diverticulitis; arteriosclerosis with hospitalization that resulted in *Staphylococcal* sepsis; limb injury as a result of an on-the-job accident with a saw that had no preceding clinical event such as dizziness, although an ongoing AE of feeling abnormal (‘brain fog’) was listed; accidental overdose resulting in encephalopathy; cervical spinal stenosis; breast cancer *in situ*; mental disorder; somatoform disorder; ovarian cyst; and chronic obstructive pulmonary disease. Serious AEs led to study withdrawal in six (1.1%) patients: one (0.2%) patient each in the SXB 4.5-g and 6-g groups, and 0 and four (3.6%) patients in the SXB 7.5-g and 9-g groups, respectively.

Given the pharmacology of SXB and prior clinical experience, specific AEs of interest are further characterized below.

#### Abuse and overdose

Patients with a history of substance abuse were excluded from the double-blind trials. Detailed review of AE preferred terms from the open-label extension study did not identify any terms consistent with abuse, dependence, or severe withdrawal during treatment. There was one case of potential misuse in which a patient enrolled at more than one study site; however, no AEs related to potential abuse were noted in this patient, and in the entire study, there was no conclusive evidence of abuse or diversion.

One case of accidental overdose was identified as a SAE (resulting in transient toxic encephalopathy); a patient who was advised to take SXB 9 g/night inadvertently took an extra 4.5 g for a total of 13.5 g for one night. The patient was hospitalized and use of the study drug was interrupted for one week, after which the patient resumed treatment and completed the study without further incident.

#### Suicidality

Patients were excluded from the double-blind trials for suicidality and major depression. There were no suicides or suicide attempts in the open-label extension; a review of AE verbatim terms, tables, and listings coded by MedDRA preferred terms, and patient-diary data identified no terms consistent with AEs related to suicidal ideation or behavior. Data from the MINI suicidality module and the BDI-II questionnaire indicated 11 patients who showed some level of suicide risk, none of which involved suicidal behavior or resulted in self-harm. Of those 11 patients, one was discontinued for a protocol violation (history of suicidality) with no other findings of potential suicidality during the trial; one was discontinued for depression but was not listed as current for either suicide risk or major depressive episode at the safety follow-up visit; one was discontinued due to rheumatoid arthritis, which developed during the double-blind period; two withdrew consent; and six completed the study.

#### Depression

Depression, considered mild or moderate in severity, was identified in 24 (4.3%) patients and led to study withdrawal in 10 patients, dose reduction in two patients and transient treatment interruption in one patient. Adjustment disorder, major depression and depressed mood were reported in 10 (1.8%) patients and were considered mild or moderate in severity. Adjustment disorder led to study withdrawal in one patient and to SXB dose reduction in another patient.

Findings either on the MINI for major depression or severe depressive symptoms on the BDI-II were reported as AEs in 16 patients. Overall mean BDI-II depression scores were decreased from baseline at all time points, and the overall percentages of patients with BDI-II scores >13 (indicative of more than minimal depressive symptoms) decreased relative to baseline (32.7% at baseline versus 16.3% at study endpoint and 12.0% at final follow-up visit; Table 3).

**Table 3:** Beck Depression Inventory version II (BDI-II) scores at baseline and endpoint

	**Baseline**	**Endpoint**
Number	559	557
Mean BDI-II score (SD)	11.1 (8.15)	5.8 (7.03)
Median BDI-II score	10.0	3.0
Range in BDI-II score	0 to 56	0 to 40
BDI-II severity (score)		
Minimal (0 to 13), number (%)	376 (67.3)	466 (83.7)
Mild (14 to 19), number (%)	97 (17.4)	62 (11.1)
Moderate (20 to 28), number (%)	67 (12.0)	21 (3.8)
Severe (29 to 63), number (%)	19 (3.4)	8 (1.4)

SD, standard deviation.

#### Respiratory adverse events including sleep-disordered breathing

The most commonly reported AE possibly related to respiratory depression was dyspnea, which was reported in 18 (3.2%) patients. Sleep apnea syndrome of moderate severity was reported in one (0.2%) patient. A diagnosis of sleep apnea in the absence of stable continuous positive airway pressure therapy was an exclusion criterion, as was an increased risk of sleep apnea that occurred during the double-blind studies.

No report of sleep-disordered breathing led to study withdrawal. Events of apnea, respiratory rate decreased and respiratory depression led to SXB dose reduction in two, one, and one patients, respectively. There were no reports of cyanosis or hypoventilation.

#### Sedation

The most common AE potentially related to CNS depression was daytime somnolence, which occurred in 34 (6.1%) patients (43 events) and was of mild or moderate severity. Somnolence led to study withdrawal in five patients and to dose reduction in 18 of the 43 events. Sedation, decreased level of consciousness, hypersomnia and hangover occurred in seven (1.3%), three (0.5%), two (0.4%) and one (0.2%) patients, respectively. Hypersomnia led to study withdrawal in one patient and to SXB dose reduction in another patient. Sedation and a lower level of consciousness each led to study withdrawal in one patient. (See also ‘Falls, syncope, and accidents’ below.)

#### Sleepwalking

Sleepwalking occurred in five (0.9%) patients; it was graded as severe but not serious in one patient and led to SXB dose reduction in two patients and study withdrawal in two patients. No sleepwalking-associated injuries were reported.

#### Falls, syncope, and accidents

Twenty (3.6%) patients had falls (potentially related to a hangover effect or to the underlying FM), which led to two study withdrawals and two patients with dose reductions. Three (0.5%) patients were involved in road traffic accidents, and two (0.4%) were involved in non-traffic accidents. While one of the traffic accidents was considered severe, none of them was deemed related to treatment, and neither of the two non-traffic accidents was related to treatment. Three patients (0.5%) had syncope, and one (0.2%) had vasovagal syncope resulting in a fall.

#### Weight changes

**Figure 3:**
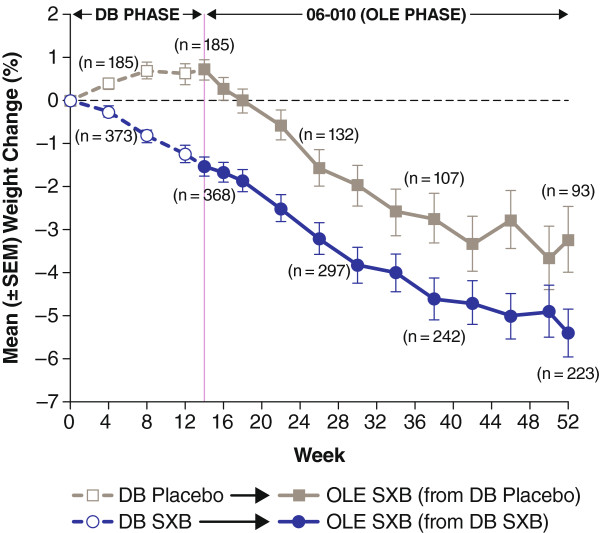
**Mean weight change from baseline for patients who entered the OLE study, by prior double-blind phase 3 study treatment.** 06**–**010 is the OLE of the DB phase 3 trials (placebo patients in the DB phase were initiated on active treatment at the start of the OLE phase). DB, double-blind; OLE, open-label extension; SEM, standard error of the mean; SXB, sodium oxybate.

A trend for decreased body weight was observed. For patients on SXB during the double-blind phase, the mean body weight continued to decline from baseline to approximately −5% by week 52 (Figure 3). A parallel reduction in body weight was observed for patients who had previously received placebo and then initiated SXB during the extension. AEs related to weight loss (decreased weight, anorexia and decreased appetite) were reported in 43 (7.7%) patients. AEs related to loss of weight were severe in one (0.2%) patient, led to SXB dose reduction in five (0.9%) patients and led to study withdrawal in four (0.7%) patients. AEs related to weight gain were less frequent; there was increased weight in two (0.4%) patients not accompanied by edema and increased appetite in one (0.2%) patient.

### Maintenance of treatment response

**Figure 4:**
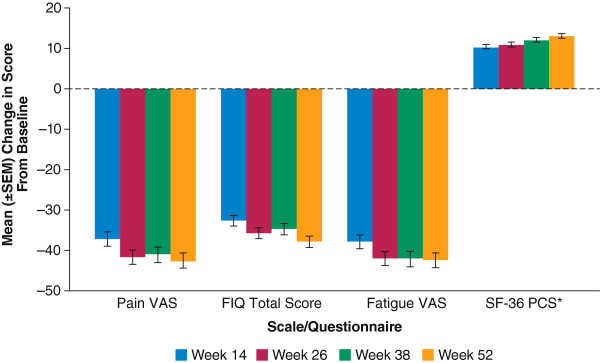
**Maintenance of sodium oxybate therapeutic response in open-label extension study for patients with at least 12 months of sodium oxybate exposure (*n*** **= 210).** *SF-36 PCS is reported for weeks 14, 18, 38 and 42. FIQ, Fibromyalgia Impact Questionnaire; SEM, standard error of the mean; SF-36 PCS, 36-item short-form health survey physical component summary; VAS, visual analog scale.

For patients with at least 12 months of SXB exposure (receiving active treatment in double-blind and open-label extension study periods combined, *n* = 210), the mean improvements from baseline observed at the end of the double-blind period (14 weeks) were maintained over the duration of the long-term extension for pain VAS, fatigue VAS, FIQ total score and SF-36 PCS (Figure 4). The mean changes from baseline to week 14 (end of the previous controlled study) and from the same controlled-study baseline to week 52 (end of the open-label extension study), respectively, were as follows: for the pain VAS, −37.2 and −42.5; for the FIQ total score, −32.6 and −37.9; and for the fatigue VAS, −37.9 and −42.3, demonstrating maintenance of effect. The mean changes from controlled-study baseline for SF-36 PCS were 10.3, 10.9, 12.1 and 13.1 points at weeks 14, 18, 38 and 52, respectively, also demonstrating maintenance of effect; changes from baseline in the MCS were minimal at all time points (data not shown).

For all treated patients in the long-term extension study, responder analyses showed that 68.8% and 53.0% achieved ≥30% and ≥50% reduction in the pain VAS at extension-study endpoint, respectively (relative to controlled-study baseline), and similar proportions achieved reductions ≥30% (69.7%) and ≥50% (52.3%) in FIQ total score (Table 4). At extension-study endpoint, 60.4% of patients responded with PGI-c responses of ‘very much better’ or ‘much better’ and investigators considered 63.2% of the patients ‘very much improved’ or ‘much improved’ in CGI-c scores (Table 4). Approximately half (50.3%) of the patients were responders in the FM Syndrome Composite Response, and 52.4% of patients were responders in the FM Pain Composite Response at the extension-study endpoint (Table 4).

**Table 4:** Responder analyses at open-label study endpoint in patients with FM syndrome

**Parameter**	**Sodium oxybate final dose**				
	**4.5 g**	**6 g**	**7.5 g**	**9 g**	**Total**^**a**^
Pain VAS	number = 165	number = 187	number = 114	number = 81	number = 551
≥30% reduction, number (%)	112 (67.9)	140 (74.9)	67 (58.8)	59 (72.8)	379 (68.8)
≥50% reduction, number (%)	88 (53.3)	110 (58.8)	47 (41.2)	47 (58.0)	292 (53.0)
FIQ total score	number = 168	number = 187	number = 114	number = 81	number = 554
≥30% reduction, number (%)	113 (67.3)	142 (75.9)	67 (58.8)	61 (75.3)	386 (69.7)
≥50% reduction, number (%)	81 (48.2)	110 (58.8)	51 (44.7)	47 (58.0)	290 (52.3)
PGI-c	number = 167	number = 187	number = 114	number = 81	number = 553
’Very much better’ or ‘much better,’ number (%)	103 (61.7)	117 (62.6)	59 (51.8)	53 (65.4)	334 (60.4)
CGI-c	number = 168	number = 187	number = 114	number = 81	number = 554
‘Very much improved’ or ‘much improved,’ number (%)	106 (63.1)	120 (64.2)	66 (57.9)	56 (69.1)	350 (63.2)
FM Syndrome Composite Response	number = 167	number = 187	number = 114	number = 81	number = 553
Responders, number (%)^b^	79 (47.3)	104 (55.6)	48 (42.1)	46 (56.8)	278 (50.3)
FM Pain Composite Response	number = 167	number = 187	number = 114	number = 81	number = 553
Responders, number (%)^c^	85 (50.9)	107 (57.2)	50 (43.9)	47 (58.0)	290 (52.4)

For Pain VAS, baseline was the average of all available daily averages during the last week of the baseline period in the double-blind studies. For post-baseline assessments, the average of all daily averages during the prior week was used. Study endpoint was defined as the last available data (study completion or early discontinuation). ^a^If the final dose was not among those indicated, the patient was summarized only for the ‘Total’ group; ^b^Fibromyalgia Syndrome Composite Responders were patients who achieved PGI-c response of ‘very much better’ or ‘much better,’ ≥30% reduction in pain VAS, and ≥30% reduction in FIQ total score at study endpoint compared with baseline of the double-blind study; ^c^Fibromyalgia Pain Composite Responders (at endpoint) were patients who achieved PGI-c response of ‘very much better’ or ‘much better’ and who had ≥30% reduction in pain VAS at study endpoint compared with baseline of the double-blind study. CGI-c, Clinical Global Impression of Change; FIQ, Fibromyalgia Impact Questionnaire; FM, fibromyalgia syndrome; PGI-c, Patient Global Impression of Change; VAS, visual analog scale.

Reduction in morning tiredness (FIQ ‘tired upon awakening’ subscale) and improvement in daytime function related to sleep (FOSQ total score) were seen across all doses (Table 5).

**Table 5:** Change from baseline at open-label study endpoint in FIQ ‘tired upon awakening’ subscale and FOSQ

**Parameter**	**Sodium oxybate final dose**				
	**4.5 g**	**6 g**	**7.5 g**	**9 g**	**Total**^**a**^
FIQ subscale: ‘tired upon awakening’					
Baseline, number	172	188	114	81	559
Mean (SD)	7.93 (1.77)	8.19 (1.74)	8.35 (1.69)	8.57 (1.48)	8.21 (1.71)
Endpoint, number	168	187	114	81	554
Mean change (SE)	−4.16 (0.25)	−4.56 (0.22)	−3.84 (0.31)	−4.88 (0.34)	−4.33 (0.13)
FOSQ total score					
Baseline, number	162	182	114	79	541
Mean (SD)	14.00 (3.82)	13.73 (3.60)	13.20 (3.61)	12.76 (3.74)	13.56 (3.71)
Endpoint, number	157	181	114	79	535
Mean change (SE)	2.75 (0.30)	3.19 (0.27)	3.28 (0.37)	3.82 (0.42)	3.15 (0.16)

Study endpoint was defined as the last available data (study completion or early discontinuation). ^a^If the final dose was not among those indicated, the patient was summarized only for the ‘Total’ group. FIQ, Fibromyalgia Impact Questionnaire; FOSQ, Functional Outcomes of Sleep Questionnaire; SD, standard deviation; SE, standard error.

## Discussion

Long-term tolerability and efficacy in FM have been observed in clinical trials with pregabalin, duloxetine, and milnacipran [13-17]. Results from this study similarly provide evidence for the tolerability of SXB during exposure of up to one year, with maintenance of clinically important improvements in pain and other FM symptoms during that period. Of the 560 patients who entered the extension after completing either of the two phase 3 double-blind studies, 402 (71.8%) patients were treated for at least six months, and 210 (37.5%) patients received treatment for at least one year; more than half (57%) of the patients who were enrolled completed the study.

There were no deaths in this study. While SAEs were reported in 20 (3.6%) patients and led to discontinuation in six of these individuals, only three SAEs in two patients were considered related to treatment: accidental overdose, transient toxic encephalopathy (resulting from the accidental overdose) and gastrointestinal hypomotility. The most frequently reported AEs were similar to those observed in previous studies of SXB in patients with narcolepsy [24] and FM [30,32,33], and nausea, anxiety and dizziness were the AEs most often resulting in study discontinuation.

There appeared to be a linear dose response for overall AEs, but not for individual AEs, although evaluation of a dose–response relationship for particular AEs was precluded by the open-label design and the allowance of flexible dosing throughout the study. However, the 9-g dose was associated with a higher incidence of nasopharyngitis, vomiting, somnolence, urinary tract infection and gastroenteritis, but there was no consistent pattern indicating that the incidence of these AEs was associated with increased doses. It should also be noted that compared with other dosage groups, the number of exposures to 9 g was lowest of all allowed dosages in the study. No abuse-related issues or signals of diversion were observed in the current study, consistent with the low levels of diversion, abuse and dependence that have been reported with use of prescribed SXB [42].

Signs of suicidality and major depression were monitored closely, especially since FM patients have been reported to have a higher prevalence of major depression than the general population [43,44], as well as an increased risk of suicide [45-47]. However, in this study, there was no sign of increased suicidality in patients receiving long-term SXB. Additionally, no risk of increased depression was detected and, although depression was not formally evaluated as an outcome, it should be noted that relative to baseline, mean BDI-II scores improved, and a smaller proportion of patients at endpoint reported depression that was greater than ‘minimal depression’ on the BDI-II.

Treatment with SXB was associated with a mean weight loss, consistent with what has been observed with SXB in a retrospective chart review of patients with narcolepsy [48]. Weight loss was reported as an AE in 3.8% of patients, with one event of decreased weight graded as severe. Of note, the mean baseline BMI for patients enrolled in this study was 28.4 kg/m^2^, a level considered pre-obese [49].

With respect to therapeutic response, SXB provided clinically important improvements over the study duration across multiple FM domains identified as important by OMERACT, including pain, functioning, fatigue and tiredness. Both patient- and investigator-rated overall well-being assessments at the end of the study indicated long-term improvement. Importantly, not only did 68.8% of patients achieve ≥30% reduction in pain compared with baseline, a clinically meaningful level of pain relief [50], but more than half (53.0%) of the patients achieved ≥50% reduction, which is considered a substantial decrease in pain [34].

The pain, fatigue, sleep-related and functional benefits were also supported by substantial improvements on the FIQ, with proportions of patients achieving the 30% and 50% thresholds similar to those observed for pain. For perspective, note that a smaller (14%) change on the FIQ has been shown to represent the minimal clinically important difference [35]. The robustness of the response across individual outcomes was also reflected by the high proportions of patients who achieved the FM Syndrome Composite Response (50.3%) and the Fibromyalgia Pain Composite Response (52.4%). Composite measures, which represent a conservative assessment of response since they require that patients fulfill two or more criteria to be considered responders, have been reported only in clinical FM trials of milnacipran [51-55] and SXB [30,32,33].

Several limitations of this study should be noted including lack of a placebo group since this was an open-label study. Common to such extension studies, patients remaining in the study generally represent a self-selected population of those who both tolerated the drug and achieved efficacy. Furthermore, this study used populations from previous clinical trials, which were restricted by specific inclusion and exclusion criteria. For both of these reasons, the results may not be fully generalizable to the clinical setting.

An additional limitation is that the overall discontinuation rate of 43% does not enable a true assessment of efficacy in all patients at the one-year time point. However, applying a worst-case scenario in which discontinued patients are assumed to be non-responders (that is, did not achieve ≥30% reduction in pain VAS) still suggests a substantial treatment benefit, with a responder rate at endpoint of 45.7%. Thus, it should be considered that the true response is likely to fall between this estimate and the 68.8% reported. It should also be noted that the overall discontinuation rate was consistent with those seen in other FM open-label extension studies [13,14,16]. The 7% discontinuations due to lack of efficacy suggests that the therapeutic effect that was maintained over the long-term and over multiple efficacy endpoints was clinically meaningful in the patients who tolerated the drug.

Overall, the long-term study results support those from the phase 2 and phase 3 controlled clinical trials [30-33] in demonstrating a similar safety profile; similar improvements across multiple clinically meaningful FM domains were observed and maintained throughout the duration of the study. The demonstrated long-term effectiveness of SXB in FM should be viewed in light of its overall safety profile and potential risks of its use.

## Conclusions

The long-term, open-label (up to 38 weeks) safety profile of SXB in FM patients was similar to those observed in the phase 2 and phase 3 double-blind trials. Treatment with SXB was associated with clinically relevant improvements across multiple FM domains, including pain and functioning; these improvements were maintained during long-term treatment (up to 52 weeks). These data support the conclusion that SXB can provide long-term benefits in the multidimensional management of FM. While multidimensional efficacy is a desired goal of therapy, any therapeutic benefits should be weighed against other factors, including individual patient needs as well as potential risks.

## Abbreviations

ACR: American College of Rheumatology; AE: adverse event; BDI-II: Beck Depression Inventory version II; BMI: body mass index; CGI-c: clinical global impression of change; CNS: central nervous system; EU: European Union; FIQ: Fibromyalgia Impact Questionnaire; FM: fibromyalgia syndrome; FOSQ: Functional Outcomes of Sleep Questionnaire; GABA: γ-aminobutyric acid; GHB: γ-hydroxybutyrate; IMMPACT: Initiatives on Methods, Measurement, and Pain Assessment in Clinical Trials; MCS: mental component summary; MedDRA: Medical Dictionary for Regulatory Activities; MINI: Mini-International Neuropsychiatric Interview; OMERACT: Outcome Measures in Rheumatology Clinical Trials; PCS: physical component summary; PGI-c: patient global impression of change; QoL: quality of life; REM: rapid eye movement; SAE: serious adverse event; SD: standard deviation; SF-36: 36-item short-form health survey; SWS: slow wave sleep; SXB: sodium oxybate; US: United States; VAS: visual analog scale.

## Competing interests

MS, SP, and IJR are consultants to and their institutions have received research support from Jazz Pharmaceuticals, Inc. MS also has acted as a consultant to Allergan and has been a consultant and participated on the speakers bureaus of Eli Lilly, Pierre Fabre Médicament, Pfizer, and UCB. CA is a consultant to UCB, Pfizer, Daiichi-Sankyo, and Grunenthal. SP acted as a consultant to Pfizer, Lilly, and Pierre Fabre Médicament. IJR has been a consultant to Pfizer, Lilly, Grunenthal, and Pierre Fabre Médicament, and has participated on the speakers bureaus of Eli Lilly and Pfizer. SAH, YGW, and DRG are employees and shareholders of Jazz Pharmaceuticals, Inc. This study was sponsored by Jazz Pharmaceuticals, Inc. The Curry Rockefeller Group, LLC, received payment from Jazz Pharmaceuticals, Inc., for editorial and graphic assistance.

## Authors’ contributions

MS, CA, IJR, YGW, SAH and DRG contributed to conception and design of the study. MS, CA, SP and IJR were involved in data acquisition. MS, CA, IJR, YGW and SAH analyzed and interpreted the data. MS, SP, IJR, YGW, DRG and SAH drafted the manuscript, with review, criticism for intellectual content, and revisions provided by all authors; all authors read and approved the final manuscript.
